# The Use of Collision Detection to Infer Multi-Camera Calibration Quality

**DOI:** 10.3389/fbioe.2015.00065

**Published:** 2015-05-12

**Authors:** Sook-Yee Chong, Beate Dorow, Ellankavi Ramasamy, Florian Dennerlein, Oliver Röhrle

**Affiliations:** ^1^SimTech Research Group on Continuum Biomechanics and Mechanobiology, Institute of Applied Mechanics (CE), University of Stuttgart, Stuttgart, Germany; ^2^Biomechatronic Systems, Fraunhofer IPA, Stuttgart, Germany

**Keywords:** error analysis, collision detection, camera calibration, accuracy, kinematics

## Abstract

Optical motion capture systems are widely used in sports and medicine. The performance of these systems depends on, amongst other factors, the quality of the camera calibration process. This study proposes a technique to assess the accuracy of the extrinsic camera parameters, as estimated during calibration. This method relies on the fact that solid objects in the real world cannot possess a gap in between, nor interpenetrate, when in contact with each other. In our study, we used motion capture to track successive collisions of two solid moving objects. The motion of solid objects was simulated based on trajectories measured by a multi-camera system and geometric information acquired from computed tomography. The simulations were then used to determine the amount of overlap or gap between them. This technique also takes into account errors resulting from markers moving close to one another, and better replicates actual movements during motion capture. We propose that this technique of successively colliding two solid moving objects may provide a means of measuring calibration accuracy.

## Introduction

Motion capture has a wide range of applications, including virtual reality, sports, and medicine. Each of these applications requires high accuracy so as to distinguish the pathological deficits from the normal, or to simulate an authentic virtual environment to train surgeons. One of the methods in motion capture is using automated three-dimensional (3D) reconstruction of moving skin markers to determine joint kinematics. A critical factor, however, affecting the accuracy of the kinematic data is the quality of the camera calibration. Errors may affect the accurate determination of joint centres, which, in turn, will affect the calculation of moments and powers at a joint.

The theory and process of deriving 3D positions of retro-reflective markers from several two-dimensional (2D) camera projections have been extensively studied (Brown, 1971; Tsai, 1987; Chen et al., 1994; Zhang, 2000). This was traditionally achieved by utilizing 2D reference points, whose 3D coordinates were then determined in a defined coordinate system. Calibration success, however, is dependent on factors such as calibration procedure, camera setup, and volume effects. A good calibration, therefore, is frequently achieved in a controlled environment such as in a gait laboratory.

In principle, accurate 3D measurements could be achieved in a conventional setting. Previous studies have reported good accuracy when commercially available camera systems were used in a predefined volume (Dorociak and Cuddeford, 1995; Ehara et al., 1997; Richards, 1999; Papic et al., 2004). Liu et al. (2007) further explored the accuracy of an optical system in the 0.5–200 μm range for really small tooth displacements. In these studies, deviations from known distances or angles between fixed markers were determined. This, however, does not reflect what happens in reality. One only has to consider the complicated movements of a multi-segmented human body to know that distances between markers are not constantly fixed while in motion.

The difficulty of calibrating multiple cameras simultaneously is increased if it was performed outdoors or in wide open spaces, where moving markers are too small for a clear and concise reconstruction and calculation of the calibration parameters (Barreto and Daniilidis, 2004). The process of being able to capture movements accurately thus continues to evolve.

In this study, we have developed a procedure for quantifying calibration accuracy of a multi-camera system based on collision detection by using markers that move relatively to each other during the calibration procedure. Saini et al. (2009) had previously utilized this procedure. In their study, the geometry of a mandibular (lower) tooth obtained from computed tomography (CT) and motion capture data of natural chewing movements were used to automatically reconstruct the tooth’s maxillary (upper) counterpart. We have, likewise, used motion capture to track successive collisions of two solid moving objects.

We simulated the recorded movement in Python programing language (https://www.python.org/), and represented the virtual objects in a voxel-based manner. One of the virtual objects served as reference, and the other as test object. In order to account for the fact that solid objects cannot penetrate each other, we progressively eroded the virtual test object by removing voxels every time it collided with the reference object. The volumetric difference between the initial and the final virtual object reflected the degree to which the simulation deviated from reality. This process, therefore, provided a measure of calibration accuracy.

## Camera Calibration

Camera calibration is the process of reconstructing the transformation from points in a world coordinate system to their corresponding points in an image plane. The transformation can be represented by a 3 × 4 matrix T_W2I_, which is composed of intrinsic and extrinsic camera parameters. Intrinsic camera parameters (focal length, image center, aspect ratio, and distortion of the lens) characterize the camera’s projection properties. Extrinsic camera parameters specify the orientation and position of the camera in the world coordinate system.

The transformation process is described by the following equation in homogeneous coordinates P_I_ = (x, y, 1) for image points and P_W_ = (X, Y, Z, 1) for real-world points:

$$\begin{array}{llll} & P_{\text{I}}\;=T_{\text{W2I}}*P_{\text{W}} & & \;\\ & \;=T_{\text{C2I}}*T_{\text{W2C}}*P_{\text{W}}\; & & \; \end{array}$$

T_W2I_ is the product of the 3 × 3 intrinsic T_C2I_ and the 3 × 4 extrinsic T_W2C_ calibration matrixes.

In the field of motion analysis, the process of finding the intrinsic parameters (linearization) and the extrinsic parameters (calibration) is performed separately. One popular technique for linearization is proposed by Zhang (2000), which takes into account projection errors by having a camera observe a planar pattern in at least two different orientations. The intrinsic parameters are then optimized for all patterns simultaneously. One technique in determining the extrinsic parameters is the wand calibration method, where a wand comprising two markers at a known fixed distance is waved in the capture volume. The 2D image coordinates attained are then used to calculate the 3D coordinates using bundle adjustment (Triggs et al., 2000).

Since the cameras in our study had already been linearized, we assumed that the intrinsic calibration parameters were known, and focused only on the camera extrinsic matrix (T_W2C_). The extrinsic parameter matrix T_W2C_ = (R|t), which consisted of a 3 × 3 rotation matrix R and a 3 × 1 translation vector t, described the camera position relative to the world coordinate system. Here, we evaluated the accuracy of T_W2C_ as estimated by the motion capture system during wand calibration.

## Methods

### Experiment

**Figure 1:**
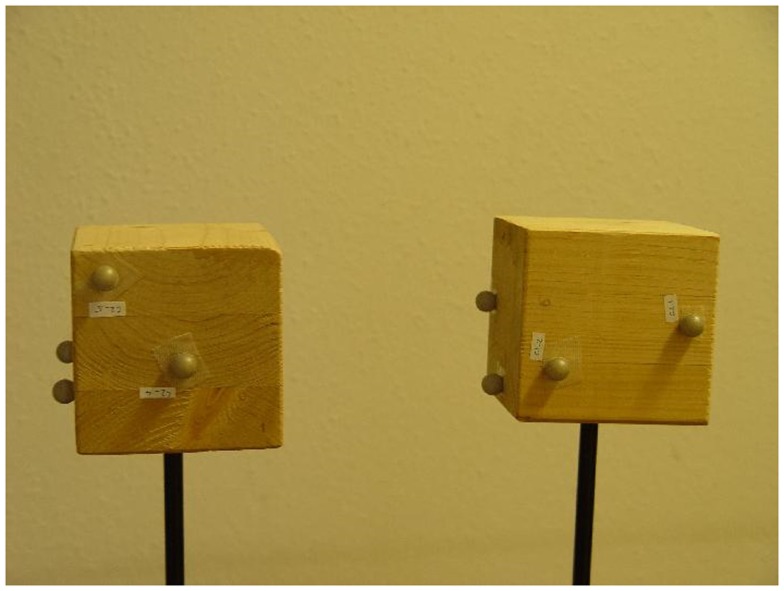
**The two cubes used in the experiment**.

We utilized two wooden cubes with lengths of 10 cm. A total of five reflective markers were randomly attached to three of the six faces of the cube. Each cube was mounted on a pole for better handling (Figure 1).

Three experiments were performed, in which the two cubes were moved relative to one another. This included random collisions and the rubbing of the marker-free faces from time to time. We ensured that entire faces were rubbed against each other. Nine cameras from Qualisys (Gothenburg, Sweden) 3D motion capture system were used to record 15 s of the marker positions at a sampling frequency of 100 Hz.

### Registration and voxelization of the cubes

A Tomoscope HV500 (Werth Messtechnik GmbH, Rudersberg, Germany) CT scan was used to capture the geometry of the cubes as a point cloud with 1.2 mm thick images. The positions of the markers’ midpoints were determined with an image processing software tool that specializes in extracting 3D regular geometric figures and spheres from point cloud data (Effenberger et al., 2013).

We computed a tight-fitting oriented bounding box around each cube’s point cloud and divided it into a 3D grid of regularly spaced voxels. The points and marker coordinates in the point cloud were then transformed to voxel indices N. Each cube was then represented as a bounding box of voxels with resolution N*N*N. Here, we assumed that each voxel is a perfect cuboid.

### Simulation

**Figure 2:**
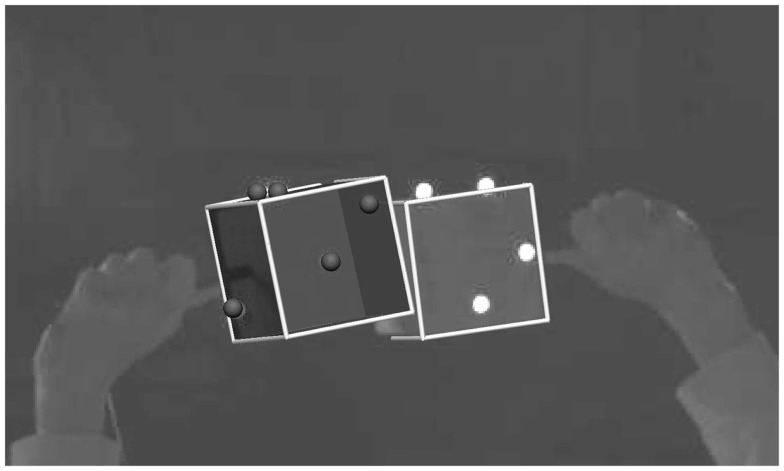
**Reconstructed experiment from markers’ trajectories**.

The experiments were simulated using Python software, with the markers’ trajectories as measured by Qualisys, and the geometric information determined from the CT scan (Figure 2). As the simulated scene mirrored the real dynamics of the cubes during the experiments, the voxel representations of the reconstructed cubes, referred to as C_R_ (reference) and C_T_ (test object), should not overlap at any time. In the simulations, however, we observed an overlap of the cubes’ surfaces at certain points in time. These overlaps occurred when they were touching or very close to each other. Given that the cube geometries had been reliably captured by the CT scan, these occurrences were ascribed to inaccurate camera calibration. The simulation might, similarly, exhibit a gap between the cubes’ faces, even though in reality the cubes were in direct contact. The two types of deviations were then categorized as Type I and Type II errors (Table 1).

**Table 1:** **Types of errors during simulation**.

Simulation	Reality	Error
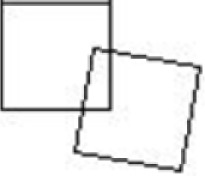	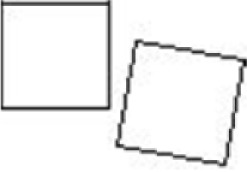 or 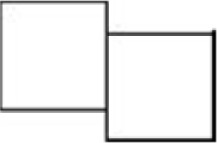	Type I
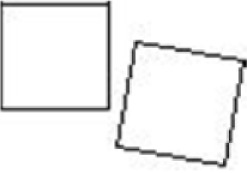	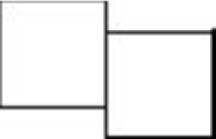	Type II

In order to measure the extent of Type I errors, the number of overlapping voxels between the two cubes was summed up for the entire time period. In order to account for Type II errors, a thin layer of voxels was added onto the contacting face of C_T_, where collision was induced during the experiments. Since we did collide and rub entire faces during the experiments, the outer voxel padding should be fully removed by the end of the simulated trial. The number of remaining outer voxels would thus indicate the magnitude of Type II error.

**Figure 3:**
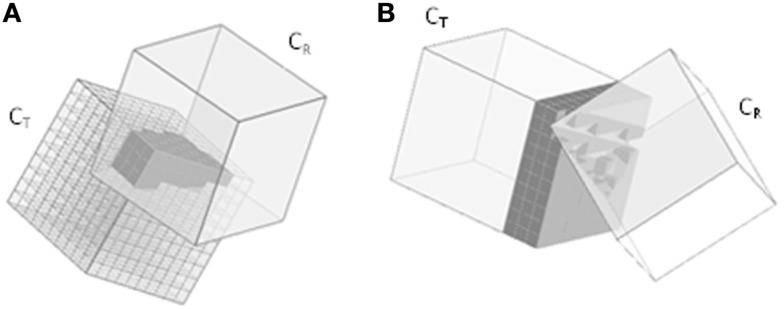
**Error detection**. **(A)** Voxelizing the cube’s interior for overlap detection (Type I). **(B)** Voxelizing the cube’s exterior for detection of spurious gaps (Type II).

In the following sections, we will use the terms “Type I voxel” and “Type II voxel” to refer to voxels representing a Type I or Type II error respectively. Figure 3 illustrates the respective procedures for detecting Type I and Type II errors.

During the simulated collisions between C_T_ and C_R_, both Type I and Type II voxels were determined. A local coordinate system with the origin specified at one corner was defined for each cube. Each voxel had 3D coordinates ranging from 1 to N, with N being the voxel resolution. The local coordinates of C_R_ were then transformed to the coordinate system of C_T_. If the transformed coordinates of a voxel fall within the coordinate system of C_T_, this is marked as a Type I voxel. In a similar manner, to determine Type II voxels, 3D coordinates ranging from N + 1 to N + d were defined for the padding, with d being the number of padding layers. If a voxel in the padding has coordinates that fall within the range of the coordinate system of C_T_, it is removed and unmarked as a Type II voxel.

**Figure 4:**
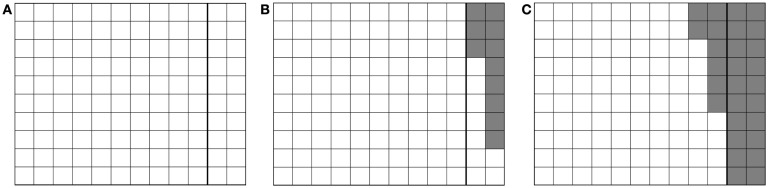
**Adjusted shape of C_T_ as time progresses**. Untouched voxels are shown in white, deleted ones in gray. The two layers of voxels on the right are the extra padding. **(A)** time t_0_ **(B)** time t_i_ **(C)** time t_j_

**Figure 5:**
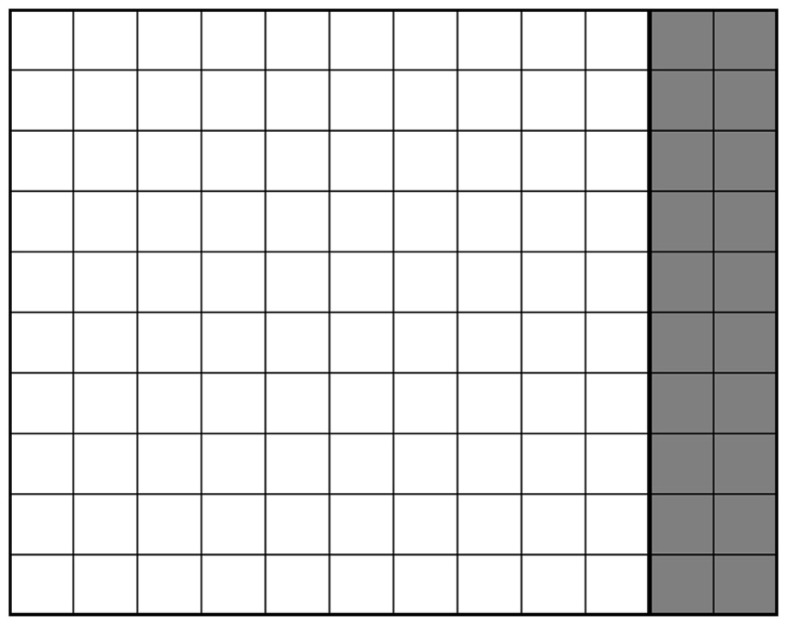
**Final shape of C_T_ in the case of a perfect simulation**.

This problem of collision detection could also be viewed as a matter of shape optimization (Saini et al., 2009). Figures 4A–C show the simplified 2D view of the adjusted shape of C_T_ at time t_0_, t_i_ and t_j_ with 0 < i < j. Here, the right side of C_T_ was in contact with C_R_. Voxels which were untouched during the simulation were shown in white; they defined the geometry of C_T_ at any given time. Deleted voxels were shown in gray. If the simulation of the cubes and their movements were perfect, the initial and final C_T_ would be identical (Figure 5).

The volumetric difference between the initial and the final shape-optimized C_T_ could be used to quantify the degree of agreement between simulation and reality. Clearly, the level of accuracy was dependent on the voxel size. The different voxel resolutions had thus been taken into account.

The total volumetric difference VolDiff_N_^(t)^ (cm^3^) at time *t* (ms) resulting from Type I and Type II errors depended on the voxel resolution of N*N*N, and was given by

(1) $${\text{VolDiff}}_{\text{N}}{\;}^{\left(\text{t}\right)}\;=({\text{n}}_{\text{I}}{\;}^{\left(\text{t}\right)}\;+\;{\text{n}}_{\text{II}}{\;}^{\left(\text{t}\right)})*{\text{v}}_{\text{N}}$$

where n_I_^(t)^ and n_II_^(t)^ are the number of Type I and Type II voxels at time t, and v_N_ is the volume of a single voxel.

## Results and Discussion

Based on the three experiments, a mean volumetric difference of 19.12 ± 5.36 cm^3^ was found (Table 2). This amounts to approximately 1.9% of the volume of C_T_. While this percentage might seem small, even tiny changes to camera calibration could affect the 3D reconstruction of markers in space (Datta et al., 2009).

**Table 2:** **Type I and II errors for all experiments at a voxel resolution of N = 128**.

	Experiment			Mean	SD
	1	2	3		
Type I	22.13	26.40	13.83	19.12	5.36
Type II	0.00	0.00	0.00	0.00	0.00

**Figure 6:**
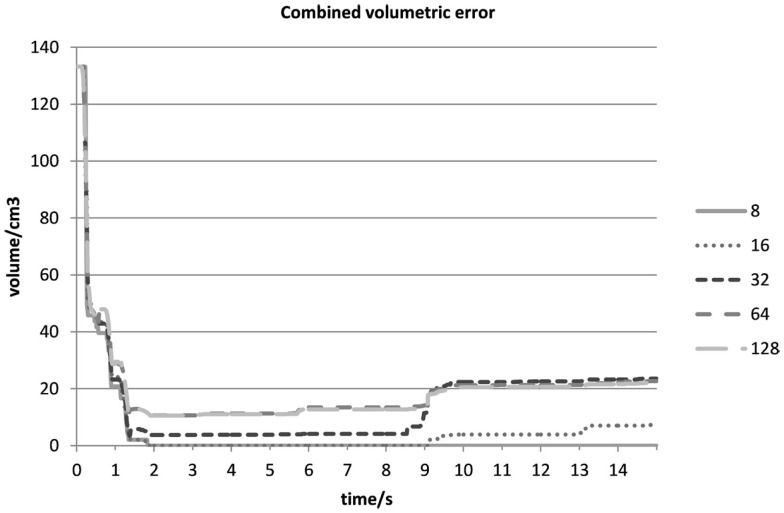
**Total volumetric difference (cm^3^) between initial and final shape-optimized C_T_ over time (s) for different voxel resolutions in Experiment 1**.

Figure 6 shows a plot of VolDiff_N_^(t)^ (cm^3^) over time (s) at varying voxel resolutions as observed in Experiment 1. At a voxel resolution of N = 8, the total volumetric difference decreased to 0 cm^3^. As mentioned, the intersection of Voxel centres between C_R_ and C_T_ was required for the removal of a voxel. For very coarse voxel resolutions (as in the case when N = 8), voxels were only removed in the case of a big discrepancy between simulation and reality. After subsequent increases in voxel resolution, we observed that the total volumetric difference converged to 22.13 cm^3^.

**Figure 7:**
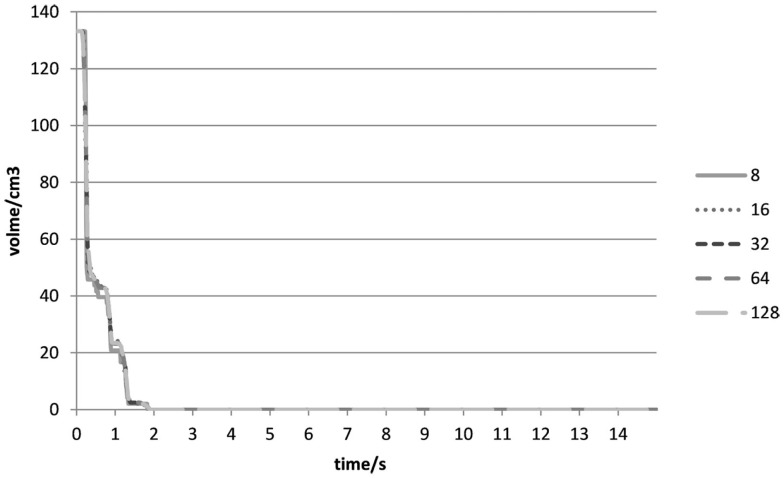
**Type II volumetric errors (cm^3^) over time (s) for different voxel resolutions in Experiment 1**.

**Figure 8:**
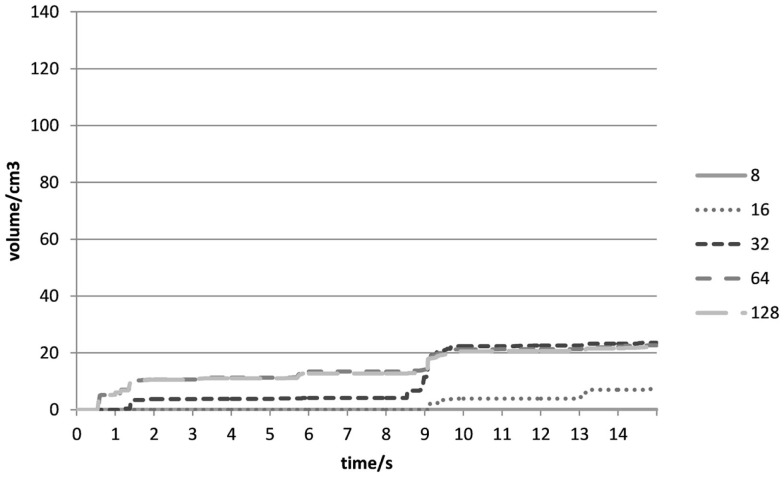
**Type I volumetric errors (cm^3^) over time (s) for different voxel resolutions in Experiment 1**.

At the beginning of the experiment (0–2 s), the sharp drop was a result of the removal of Type II voxels (Figures 3B and 7) when both cubes were in contact with each other. It would seem that no gaps were found between the cubes in all the experiments, and Type II voxels were not as problematic as had been anticipated. As all the outer voxels had been removed, subsequent increases in the combined volumetric error were due to Type I voxels (Figure 8) being removed at instances when both cubes were rubbing against each other.

For this technique to work, therefore, an appropriate voxel resolution had to be chosen carefully so as to determine the volumetric difference between the initial and final-shaped C_T_. In our study, a voxel resolution of N > 64 demonstrated a convergence to the total volumetric difference.

In motion capture systems, the determination of the intrinsic and extrinsic parameters is performed separately. In our study, we evaluated the accuracy of the extrinsic parameter matrix during wand calibration, assuming that the intrinsic parameters were already known. Our proposed collision detection technique could also be utilized when intrinsic parameters are unknown or to be determined simultaneously with the extrinsic parameters. Larger errors, however, will be expected. This is because while it is simpler to use the wand calibration method to simultaneously determine both intrinsic and extrinsic parameters, all the parameters will ultimately become sensitive to the movement of the calibration wand^1^. This may, therefore, result in larger calibration errors.

It is clear that there may be other sources of errors affecting the extrinsic calibration parameters, such as camera placement. In our study, average residuals of 0.46–0.75 mm were recorded during calibration, which were within the accepted range of 0.5–1.5 mm as recommended by Qualisys. It is, therefore, apparent that while intrinsic parameters such as lens distortion cannot be completely eradicated, random errors could still be determined and minimized. In a controlled environment such as in the laboratory, high quality calibration could easily be achieved. In measurements performed outdoors and/or where large capture volumes are required, it becomes difficult to consider all the 2D image coordinates simultaneously so as to attain the 3D coordinates required to determine the extrinsic camera parameters (Barreto and Daniilidis, 2004). We are not suggesting that this technique should replace the commonly used calibration procedures (recommended by Qualisys and/or other motion capture systems), but our approach can instead complement these procedures to minimize calibration errors.

This technique could be an additional criterion to the wand calibration method, since this collision detection takes into account the errors that resulted from markers moving close to one another. The wand calibration method includes waving a wand with two markers at a fixed distance in the capture volume. Previous studies performing accuracy tests of several motion capture systems also utilized markers at fixed distances to each other (Dorociak and Cuddeford, 1995; Ehara et al., 1997; Richards, 1999; Papic et al., 2004). Compared to our technique, the use of fixed markers in the previous studies was straightforward and easier to implement. Distances between moving markers, however, are not constantly fixed during capture. The human body, consisting of multi-linkages, is fully capable of performing complicated 3D movements. Our technique, therefore, better replicates actual movements during motion capture.

In conclusion, we have proposed a procedure based on collision detection, which could be used as an indicator of calibration accuracy. This technique can complement current calibration methods to minimize calibration errors when simultaneous calibration of multiple cameras is required.

## Conflict of Interest Statement

The authors declare that the research was conducted in the absence of any commercial or financial relationships that could be construed as a potential conflict of interest.

## Footnotes

^1^Hofverberg, S. (2007). Theories for 3D reconstruction. Qualisys Technical Note.
