# Erratum: The Use of Collision Detection to Infer Multi-Camera Calibration Quality

**DOI:** 10.3389/fbioe.2015.00134

**Published:** 2015-08-31

**Authors:** Frontiers Production Office

**Affiliations:** ^1^Frontiers Production Office, Frontiers, Switzerland

**Keywords:** error analysis, collision detection, camera calibration, accuracy, kinematics

## Reason for Erratum

Due to an oversight, the name of the author Oliver Röhrle was published as Oliver Röehrle instead of Oliver Röhrle. This mistake does not change the scientific conclusions of the article in any way.

The publisher apologizes for this error.

Original article has been updated.

